# Supplementary material for: Obesity and eating disorders in integrative prevention programmes for adolescents: protocol for a systematic review and meta-analysis
Source: BMJ Open. 2018 Apr 19;8(4):e020381. doi: 10.1136/bmjopen-2017-020381 (PMC5914714; doi:10.1136/bmjopen-2017-020381)
Supplement: Supplementary file 1 [file bmjopen-2017-020381supp001.pdf]

Supplement figure 1 – Search strategy in MedLine

exp "Feeding and eating disorders"/	27,825
(bing* and (food or eat*)).ti.	1,963
(bing* and (food or eat*)).ab.	5,375
(bing* and (food or eat*)).kw.	199
(disorder* and (food or eat*)).ti.	9,465
(disorder* and (food or eat*)).ab.	28,521
(disorder* and (food or eat*)).kw.	72
(anorexia or anorexic).ti,ab,kw.	28,138
bulimi* or bulemi*).ti,ab,kw.	7,825
1 or 2 or 3 or 4 or 5 or 6 or 7 or 8 or 9	63,512
exp Obesity/	183,262
exp Overweight/	188,264
exp Body Weight/	422,550
obes*.ti,ab,kw.	247,930
(overweight or "over weight").ti,ab,kw.	55,791
11 or 12 or 13 or 14 or 15	528,049
exp Primary Prevention/	138,711
"prevention & control".fs.	1,201,380
exp Health Promotion/	68,683
exp Health Education/	156,404
exp School Health Services/	22,370
prevent*.ti,ab,kw.	1,207,081
educat*.ti,ab,kw.	497,253
promot*.ti,ab,kw.	812,498
17 or 18 or 19 or 20 or 21 or 22 or 23 or 24	3,303,119
exp Adolescent/	1,884,302
(adolescen* or teen* or youth*).ti,ab,kw.	296,306
26 or 27	1,955,018
10 and 6 and 25 and 28	982
