# Supplementary material for: Obesity and eating disorders in integrative prevention programmes for adolescents: protocol for a systematic review and meta-analysis
Source: BMJ Open. 2018 Apr 19;8(4):e020381. doi: 10.1136/bmjopen-2017-020381 (PMC5914714; doi:10.1136/bmjopen-2017-020381)
Supplement: Author's manuscript [file bmjopen-2017-020381.draft_revisions.pdf]

BMJ Open is committed to open peer review. As part of this commitment we make the peer review history of every article we publish publicly available.

When an article is published we post the peer reviewers' comments and the authors' responses online. We also post the versions of the paper that were used during peer review. These are the versions that the peer review comments apply to.

The versions of the paper that follow are the versions that were submitted during the peer review process. They are not the versions of record or the final published versions. They should not be cited or distributed as the published version of this manuscript.

BMJ Open is an open access journal and the full, final, typeset and author-corrected version of record of the manuscript is available on our site with no access controls, subscription charges or pay-per-view fees (http://bmjopen.bmj.com).

If you have any questions on BMJ Open's open peer review process please email info.bmjopen@bmj.com

BMJ Open

Obesity and eating disorders in integrative prevention programs for adolescents: protocol for a systematic review and meta-analysis

Journal:	*BMJ Open*
Manuscript ID	bmjopen-2017-020381
Article Type:	Protocol
Date Submitted by the Author:	03-Nov-2017
Complete List of Authors:	Barco Leme, Ana Carolina; Universidade de Sao Paulo, Nutrition ; Baylor College of Medicine, Children's Nutrition Research Center<br>Thompson, Debbe ; Baylor College of Medicine, Children's Nutrition Research Center<br>Lenz Dunker, Karin ; Universidade Federal de Sao Paulo Escola Paulista de Medicina, Department of Psychiatric<br>Nicklas, Theresa; Baylor College of Medicine, Children's Nutrition Research Center<br>Tucunduva Philippi, Sonia ; Universidade de Sao Paulo<br>Lopez, Tabbetha ; University of Houston College of Liberal Arts and Social Sciences, Health and Human Performance<br>Vézina-Im, Lydi-Anne; Baylor College of Medicine, Pediatrics-Nutrition<br>Baranowski, Tom; Baylor College of Medicine, Department of Pediatrics
Keywords:	Adolescent, obesity prevention, Eating disorders < PSYCHIATRY, systematic review, disordered eating

SCHOLARONE™
Manuscripts

Obesity and eating disorders in integrative prevention programs for adolescents: protocol for a systematic review and meta-analysis

Ana Carolina Barco Leme[1,2]; Debbe Thompson[2], Karin Louise Lenz Dunker[3], Theresa Nicklas[2], Sonia Tucunduva Philippi, Tabbetha D. Lopez[4], Lydi-Anne Vézina-Im[1]; Tom Baranowski[2]

[1] School of Public Health, University of São Paulo, São Paulo, Brazil
[2] Children's Nutrition Research Center, Baylor College of Medicine, Houston, Texas
[3] Department of Psychiatry, Federal University of São Paulo, São Paulo, Brazil
[4] Department of Health and Human Performance, University of Houston, Houston, Texas

E-mails:
Ana Carolina Barco Leme acarol.leme@gmail.com
Debbe Thompson Deborah.Thompson@ARS.USDA.gov
Karin Lousie Lenz Dunker kdunker00@yahoo.com
Theresa Nicklas tnicklas@bcm.edu
Sonia Tucunduva Philippi philippi@usp.br
Tabbetha D. Lopez tabbethalopez@gmail.com
Lydi-Anne Vézina-Im Lydi-Anne.Vezina-Im@bcm.edu
Tom Baranowski tbaranow@bcm.edu

Corresponding author: Ana Carolina Leme
Corresponding author address: Children Nutrition Research Center, Baylor College of Medicine. 1100 Bates Street, Houston, Texas, USA 77003

Abstract for protocol

Introduction: Obesity and eating disorders (ED) are public health problems that have lifelong financial and personal costs, and common risk factors, e.g., body dissatisfaction, weight teasing and disordered eating (DE). Obesity prevention interventions might lead to the development of an ED since focusing on weight in

For peer review only

addition to being ineffective in weight loss, can contribute to excessive concern with diet and weight. Therefore, the proposed research will assess whether integrating obesity and ED prevention procedures do better than single approach interventions in preventing obesity among adolescents, and if integrated approaches influence weight-related outcomes. **Methods and analysis**: Integrated obesity and ED prevention interventions will be identified. Randomized controlled trials reporting data on adolescents ranging from 10 to 19 years of age from both sexes will be included. Outcomes of interest include body composition, unhealthy weight control behaviors, and body satisfaction measurements. MEDLINE/PubMed, PsycINFO, Web of Science and SciELO will be searched. Data will be extracted independently by two reviewers using a standardized data extraction form. Trial quality will be assessed using the Cochrane Collaboration criteria. The effects of integrated vs. single approach intervention studies will be compared using systematic review procedures. If an adequate number of studies report data on integrated interventions among similar populations (k>5), a meta-analysis with random-effects will be conducted. Sensitivity analyses and meta-regression will be performed only if between-study heterogeneity is high ($I^2 \geq 75\%$). **Ethics and dissemination**: Ethics approval will not be required as this is a systematic review of published studies. The findings will be disseminated through conference presentations and peer-reviewed journals.

For peer review only

Strengths and Limitations

- Systematic review and meta-analysis of randomized controlled trials
- According to PRISMA-P statement
- Registered in PROSPERO (CRD42017076547)
- Body composition will be objective measures
- Disordered eating will be assessed through self-reported measurements

Keywords: Adolescent, obesity prevention, eating disorders, systematic review, disordered eating

Introduction

Pediatric overweight and obesity are worldwide public health concerns[1], with the highest rates in the USA where 28.8% of boys and 29.7% of girls are overweight or obese[2]. Western low- and middle-income countries (LMIC) also face unhealthy child weight, e.g., 24.3% of individuals between 10-19 years of age in Brazil were overweight or obese[2]. Some evidence indicates a rapid increase in prevalence levels in LMICs as high or even higher than those found in high-income countries (HICs)[3]. Obesity has been associated with long and short-term physical health conditions, such as cardio-metabolic diseases[4], certain types of cancers[5], and mental health concerns[6,7]. Overweight youth are also at risk of becoming obese adults[8], indicating prevention should be initiated in youth.

Prior systematic reviews have examined childhood obesity prevention studies [3,9,10]. Findings, however, have been mixed. In one review of school-based interventions preventing obesity among children and adolescents, an average difference between the intervention and control groups was -0.33kg/m^2 (-0.55, -0.11, 95%CI), with 84% of this effect explained by the highest quality studies[11]. Alternatively, another reported a difference of 0.03 (95%CI: 0.09 to 0.03, p=0.03) with high heterogeneity (I^2=87%) [12]. Thus, evidence regarding the effectiveness of school-based obesity prevention interventions to reduce BMI in youth is mixed with high heterogeneity among studies. More narrow age groups who experience common problems and receive interventions appropriate to these common problems may be more effective.

Eating disorders (ED) are illnesses in which the people experience severe disturbances in their eating behaviors and related thoughts and emotions. People with ED typically become pre-occupied with food and their body weight[13]. In DSM-5, the ED section was renamed “Feeding and Eating Disorders” and specified three ED’s: anorexia nervosa (AN), bulimia nervosa (BN) and binge eating disorder (BED); and three feeding disorders: pica, rumination disorder, and avoidant/restrictive food disorder[13]. These categories, and associated criteria served to decrease the frequency of the diagnostic category “eating disorder not otherwise specified” (EDNOS), a heterogeneous not well-defined group of ED. EDNOS was the most common diagnosis in clinical and community samples of adolescents, accounting for around 80% of all ED diagnoses, with psychopathology and adverse consequences comparable to AN and BN[13,14].

For peer review only

Due to the increasing prevalence of obesity and ED[15 16] and shared common risk factors, i.e., body dissatisfaction, unhealthy weight control behaviors/dieting and weight teasing (Figure 1) there have been calls for integration to address these common concerns[15]. For instance, obesity and ED can co-occur in the same individual[15]. A cross-cultural comparison between US and Spanish adolescents found dieting and use of unhealthy weight control behaviors were higher among overweight and obese youth and concluded that prevention interventions should address the broad spectrum of eating and weight-related problems[17].

Disordered eating (DE) behaviors and attitudes are part of the ED continuum and include obsessively thinking about food and calories, becoming angry when hungry, being unable to select what to eat, seeking food to compensate for psychological problems, eating until feeling sick, and presenting unreal myths and beliefs about eating and weight[18]. DE is not limited to those diagnosed with ED. Indeed, many individuals experience DE habits, beliefs, and feelings toward food but are unaware that they are manifesting "abnormal" behaviors[19].

Being overweight during childhood increases the chances of having an ED during adulthood (compared to normal weight controls) [17]. Some interventions have addressed both obesity and DE in prevention interventions because of the efficiency in addressing two conditions with a single intervention and a possible reduced risk of inadvertently causing ED while trying to prevent obesity[15,20,21], e.g., strategies to prevent obesity (monitoring intake and portion control) might unintentionally promote shape concerns and DE. Integrating obesity and ED prevention programs may prove easier and more cost-effective than treating them separately, and healthy nutrition and physical activity are the focus of both ED and obesity prevention programs[22]. Body dissatisfaction concerns are also addressed in both approaches, but have mismatched messages. For example, some obesity prevention programs consider it acceptable to be unhappy about being overweight in order to motivate restricting the amount and content of food consumed to reduce body weight[22], while ED prevention programs promote self-acceptance at any weight, discouraging self-consciousness about dietary intake. However, data supporting these alternative explanations are scarce [22].

Common obesity and ED risk factors can be categorized into three levels according to the Social Ecological Model (SEM)[23]: individual (e.g., sex, age and weight-status), social (e.g., media, weight teasing and ideals beauty pattern) and psychological (e.g., self-esteem and body satisfaction). Several studies have described

For peer review only

the co-presence of these factors, considered risks for the development of an ED and/or obesity[24-26]. Thus, an integrated approach needs to address the differences in these prevention philosophies, e.g., eating behaviors (dieting vs. no dieting) and body weight (lose vs. accept weight)[27].

Few programs aimed at preventing ED assessed the impact on weight status and other obesity-related outcomes[16,28]. Programs included content of relevance to obesity prevention (e.g., promotion of healthy weight management). More recent prevention programs focused on protective factors such as life skills and emotion regulation competence, with the risk for ED reduced. [27]

In summary, obesity and ED have common risk factors with diverse negative health outcomes, mainly among overweight and female adolescents. Systematic reviews and meta-analyses have only analyzed results for single approaches (i.e., obesity or ED prevention) and the results have been mixed. Interventions that integrate obesity and ED prevention components might be more effective. A review of such interventions might provide insight into the mechanisms of effect and inform interventions that address both problems simultaneously. To the authors' knowledge, no previous review has identified the impact of integrated obesity and ED prevention programs for adolescents. The present systematic review will answer the following questions:

- Do integrated programs do better than obesity-only prevention programs in improving adolescents' health behavior outcomes and maintaining healthy weight status?
- Do integrated interventions promote being more satisfied with one's body and reduce unhealthy weight control behaviors in adolescents?

Methods and analysis

The study protocol was accepted in PROSPERO (www.crd.york.ac.uk/PROSPERO) in October 2017 (CRD42017076547). This protocol follows the Preferred Reporting Items for Systematic Review and Meta-Analysis Protocol (PRISMA-P) checklist[29]. Modifications to the protocol will be tracked and dated in PROSPERO.

For peer review only

Criteria for considering studies for this review

Inclusion criteria

Population: adolescents 10 to 19 years of age from both sexes. Adolescents in this age range are at increased risk for unhealthy weight control behaviors and body satisfaction, shared risk factors for obesity and ED. Most published integrated prevention studies are in this age group.

Type of outcomes: (1) body composition measurements (i.e., body mass index (BMI), waist circumference or percent body fat); (2) weight control behaviors and/or scales that assess the risk for an ED (such as the Eating Attitudes Test (EAT-26), Sociocultural attitudes towards appearance questionnaire 3 (SATAQ-3) and Eating Disorder Examination Questionnaire (EDE-Q)); (3) self-reported scales on body satisfactions and; (4) other psychological markers (e.g., anxiety, depression and/or self-esteem inventories). Inclusion of at least one of the weight control behaviors and/or scales must have been used to assess the risk for ED [19,27,30,31].

Study design: randomized controlled trials assessing the impact of integrated or obesity- only prevention interventions.

Type of studies: Quantitative outcome analyses will be included in the systematic review and meta-analysis.

Search strategy

A structured electronic search will employ all publication years (through July 2017) using four databases: Medical Literature Library of Medicine (MEDLINE) via PubMed (≥1979), PsycINFO of the American Psychological Association (≥1954), Web of Science via Clarivate Analytics (≥1983) and Scientific Electronic Library (SciELO) via BIREME Latin American and Caribbean Center on Health Science Information (≥1997). Systematic searchers will be developed from this model, applied in MEDLINE: (Obesity) OR Overweight) OR Weight related problems) AND DE) OR ED) OR weight control behaviors) AND adolescents) OR youth) OR teenagers) OR girls) OR boys) AND prevention) OR strategies) OR randomized controlled trial. Congress abstracts, dissertations, theses, and articles published in journals without peer-review will not be included in the review. Only studies written in English, German, Spanish or Portuguese will be included. The results of this search strategy will be reported in a PRISMA flowchart[29]. The bibliographies of papers that match

For peer review only

inclusion criteria will be searched by hand to identify further relevant references, which will be subjected to the same screening and selection process.

Screening and data extraction

All articles identified from the initial electronic search process will be imported into an Endnote library, and duplicates removed. The eligibility criteria will be applied to the results and all identified references screened independently by two reviewers (AL and TL) in a standard blinded way in four stages: (i) reviewing the titles and abstracts; (ii) retrieving and examining the full texts for inclusion; (iii) searching references lists from the full articles; and (iv) examining relevant references for additional studies. TB will be consulted when questions or ambiguity arise. The data extraction form will be pre-tested with 5 randomly selected trials.

Quality assessment

The quality of the randomized controlled trials will be assessed using the Cochrane Collaboration's tool for assessing risk of bias in randomized trials [32]. All data will be extracted and quality assessed by two reviewers. Disagreements at each step will be resolved by discussion. When no consensus is reached a third reviewer will resolve the discrepancy.

Data synthesis and analysis

The results of the studies included in the systematic review will be described in a summary table, consisting of author (year), purpose of the study, population targeted, study quality[32], characteristics of the sample, outcome measures, statistical analyses performed (e.g., repeated measure ANOVA, ANCOVA or regression analysis) and the results on body composition and DE behaviors. The results of the impact of the intervention will be reported in effect sizes, such as Odd Ratios (OR) for dichotomous outcomes (e.g., satisfied and dissatisfied) or standardized mean differences (SMD) for continuous outcomes (e.g., BMI – Kg/m^2). All effect sizes will be zero order. To facilitate interpretation and permit comparison with other SMD and standard effect sizes, the ORs will be converted to Cohen's d[33]. Cohen's d of 0.2 is a small effect size, 0.5 is medium and ≥0.80 is large[33].

An adequate number of studies ($k>5$)[34] will trigger a meta-analysis of the findings with a random-effects model. The magnitude of the effect sizes might vary

For peer review only

across the studies due to the differences in sample and outcomes of the studies. The pooled effect sizes will be computed, and each study will be weighted according to its sample size. Cochran's Q[35] and I^2 statistics[36] will assess the between-study heterogeneity, as measures of the percentage of total variation in estimated effects that is a consequence of heterogeneity rather than chance[37]. Significant heterogeneity is considered when the Q statistic has $p<0.05$. An I^2 statistic of 25% or less is considered low; 50% moderate and 75% high heterogeneity[38]. If study heterogeneity exceeds $I^2 \geq 75\%$ (high), it will be explored through sensitivity analyses and meta-regression. The funnel plot will be inspected for publication bias, with a minimum of 10 studies in the analysis[39], through Duval and Tweedie's trim and fill method[40] and Egger's regression test[41]. All the analyses will be conducted using Comprehensive Meta-Analysis software for Mac.

Subgroup analyses might be conducted to assess the possible effects of time differences between integrated prevention versus single obesity approach, and between certain DE behaviors and anthropometric measurements according to the following variables: population (e.g., normal weight adolescents vs. overweight/obese adolescents) and quality rating (high-rated vs. low-rated studies according to the Cochrane Collaboration's tool[32]). Age and sex differences in the impact of integrated prevention programs will be examined since DE behaviors are more common among older adolescents, girls and overweight/obese individuals[15,42,43]. Moreover, because previous studies[44-47] have found socioeconomic disparities in obesity and DE socioeconomic status, differences will be examined in ED and obesity risk factors in the prevention conditions.

The strength of the evidence will be evaluated using the Grading of Recommendations Assessment, Development and Evaluation (GRADE) guidelines[48]. The following assessments will be made: (1) Quality rating for each study according to Cochrane Collaboration's tool[32]; (2) Cohen's d classification to evaluate the magnitude of individual or pooled effect size (SMD)[33], if a meta-analysis is possible; (3) Cochran's Q[35] and I^2 statistic[36] for heterogeneity; and (4) risk of bias by visualizing the distribution of the funnel plot if there are at least 10 trials per analysis[39] through Duval and Tweedie's trim and fill method and Eggers's regression test.

Gaps and limitations

For peer review only

Several gaps and limitations should be noted in anticipation of the findings of the systematic review and meta-analysis. First, the body composition measurements reported in these studies will always be objective measurements (i.e., BMI, waist circumference and %body composition) which do not precisely measure percent body fat[49-51]. Second, DE will be assessed through self-reported measurements which might provide biased responses, since underreporting is highly prevalent, especially among girls and overweight/obese individuals[42,46,47,52,53]. Any studies that assess health preventive intervention impacts on self-reported or anthropometric data may potentially underestimate the effect of the intervention.

Implications

This systematic review and meta-analysis aims to evaluate the impact of obesity and ED prevention programs for adolescents. Results of this study should provide new insights into the approaches tested thus far. The systematic review and meta-analysis may also identify specific gaps in the evidence, which would inform the agenda for future research and policy.

Amendments

If there is a need to amend this protocol, the date, rationale, and a description of each protocol change will be reported.

Ethics and dissemination

Ethics approval will not be required as this is a protocol for systematic review and meta-analysis. This systematic review and meta-analysis will be published in a peer-reviewed journal which will be disseminated electronically and in print.

Abbreviations

DE: disordered eating; DSM-V: Diagnostic and Statistical Mental disorders 5th edition; ED: eating disordered; EDNOS: Eating Disorders Not Otherwise Specified; EPHPP: Effective Public Health Project; GRADE: Grading of Recommendations Assessment Development and Evaluation; HIC: High-income countries; LMIC: Low-middle-income countries.

For peer review only

References

1. Hoelscher DM, Kirk S, Ritchie L, Cunningham-Sabo L, Academy Positions C. Position of the Academy of Nutrition and Dietetics: interventions for the prevention and treatment of pediatric overweight and obesity. *Journal of the Academy of Nutrition and Dietetics.* 2013;113(10):1375-1394.
2. Ng M, Fleming T, Robinson M, et al. Global, regional, and national prevalence of overweight and obesity in children and adults during 1980–2013: a systematic analysis for the Global Burden of Disease Study 2013. *The Lancet.* 2014;384(9945):766-781.
3. Verstraeten R, Roberfroid D, Lachat C, et al. Effectiveness of preventive school-based obesity interventions in low- and middle-income countries: a systematic review. *The American journal of clinical nutrition.* 2012;96(2):415-438.
4. Moreno LA. Obesity in children and adolescents. A critical review. *Endocrinología y Nutrición.* 2013;60:7-9.
5. Kann L, McManus T, Harris WA, et al. Youth Risk Behavior Surveillance - United States, 2015. *MMWR Surveill Summ.* 2016;65(6):1-174.
6. Reilly JJ, Kelly J. Long-term impact of overweight and obesity in childhood and adolescence on morbidity and premature mortality in adulthood: systematic review. *Int J Obes (Lond).* 2011;35(7):891-898.
7. Wake M, Canterford L, Patton GC, et al. Comorbidities of overweight/obesity experienced in adolescence: longitudinal study. *Archives of disease in childhood.* 2010;95(3):162-168.
8. Tsiros MD, Coates AM, Howe PR, Grimshaw PN, Buckley JD. Obesity: the new childhood disability? *Obesity reviews : an official journal of the International Association for the Study of Obesity.* 2011;12(1):26-36.
9. Wang Y, Cai L, Wu Y, et al. What childhood obesity prevention programmes work? A systematic review and meta-analysis. *Obes Rev.* 2015;16(7):547-565.
10. Boff RM, Liboni RPA, Batista IPA, de Souza LH, Oliveira MDS. Weight loss interventions for overweight and obese adolescents: a systematic review. *Eating and weight disorders : EWD.* 2017;22(2):211-229.
11. Silveira JA, Taddei JA, Guerra PH, Nobre MR. The effect of participation in school-based nutrition education interventions on body mass index: a meta-analysis of randomized controlled community trials. *Preventive medicine.* 2013;56(3-4):237-243.
12. Guerra PH, Nobre MR, da Silveira JA, Taddei JA. School-based physical activity and nutritional education interventions on body mass index: a meta-analysis of randomised community trials - project PANE. *Preventive medicine.* 2014;61:81-89.
13. APA. *Diagnostic and Statistical Manual of Mental Disorders (DSM-5®).* Arlington, VA: American Psychiatric Association;2013.
14. Smink FR, van Hoeken D, Oldehinkel AJ, Hoek HW. Prevalence and severity of DSM-5 eating disorders in a community cohort of adolescents. *Int J Eat Disord.* 2014;47(6):610-619.
15. Haines J, Neumark-Sztainer D. Prevention of obesity and eating disorders: a consideration of shared risk factors. *Health Educ Res.* 2006;21(6):770-782.

For peer review only

16. Leal GVS, Philippi ST, Polacow VO, Cordas TA, Alvarenga Mdos S. O que é comportamento de risco para transtornos alimentares em adolescentes? *Jornal Brasileiro de Psiquiatria.* 2013;62 (1):62-75.

17. Lopez-Guimera G, Neumark-Sztainer D, Hannan P, Fauquet J, Loth K, Sanchez-Carracedo D. Unhealthy weight-control behaviours, dieting and weight status: a cross-cultural comparison between North American and Spanish adolescents. *Eur Eat Disord Rev.* 2013;21(4):276-283.

18. Alvarenga Mde F, Scagliusi F, Philippi ST. Development and validity of the Disordered Eating Attitude Scale (DEAS). *Perceptual and motor skills.* 2010;2010(110):379-395.

19. Alvarenga Mdos S, Scagliusi FB, Philippi ST. Development and validity of the Disordered Eating Attitude Scale (DEAS). *Percept Mot Skills.* 2010;110(2):379-395.

20. Tanofsky-Kraff M, Shomaker LB, Wilfley DE, et al. Targeted prevention of excess weight gain and eating disorders in high-risk adolescent girls: a randomized controlled trial. *Am J Clin Nutr.* 2014;100(4):1010-1018.

21. Sanchez-Carracedo D, Fauquet J, Lopez-Guimera G, et al. The MABIC project: An effectiveness trial for reducing risk factors for eating disorders. *Behav Res Ther.* 2016;77:23-33.

22. Irving LM, Neumark-Sztainer D. Integrating the prevention of eating disorders and obesity: feasible or futile? *Preventive medicine.* 2002;34(3):299-309.

23. Sallis JF, Owen N, Fisher EB. Ecological models of health behavior, in Health behavior and health education. Theory, research, and practice. . In: San Francisco, CA: Jossey-Bass; 2008:465–485.

24. Balantekin KN, Birch LL, Savage JS. Family, friend, and media factors are associated with patterns of weight-control behavior among adolescent girls. *Eat Weight Disord.* 2017.

25. Frederick DA, Sandhu G, Scott T, Akbari Y. Reducing the negative effects of media exposure on body image: Testing the effectiveness of subvertising and disclaimer labels. *Body image.* 2016;17:171-174.

26. Loth K, Wall M, Larson N, Neumark-Sztainer D. Disordered eating and psychological well-being in overweight and nonoverweight adolescents: secular trends from 1999 to 2010. *The International journal of eating disorders.* 2015;48(3):323-327.

27. Sanchez-Carracedo D, Neumark-Sztainer D, Lopez-Guimera G. Integrated prevention of obesity and eating disorders: barriers, developments and opportunities. *Public health nutrition.* 2012;15(12):2295-2309.

28. Stice E, Shaw H, Marti CN. A meta-analytic review of eating disorder prevention programs: encouraging findings. *Annu Rev Clin Psychol.* 2007;3:207-231.

29. Moher D, Liberati A, Tetzlaff J, Altman DG, Group P. Preferred reporting items for systematic reviews and meta-analyses: the PRISMA statement. *PLoS medicine.* 2009;6(7):e1000097.

30. Neumark-Sztainer D. Integrating messages from the eating disorders field into obesity prevention. *Adolesc Med State Art Rev.* 2012;23(3):529-543.

31. Neumark-Sztainer D. Addressing obesity and other weight-related problems in youth. *Arch Pediatr Adolesc Med.* 2005;159(3):290-291.

32. Higgins JP, Altman DG, Gotzsche PC, et al. The Cochrane Collaboration's tool for assessing risk of bias in randomised trials. *BMJ.* 2011;343:d5928.

33. Cohen J. A power primer. *Psychol Bull* 1992;112:155–159.

For peer review only

34. Davey J, Turner RM, Clarke MJ, Higgins PT. Characteristics of meta-analyses and their component studies in the Cochrane Database of Systematic Reviews: a cross-sectional, descriptive analysis. *BMC public health.* 2011;11.
35. Cochran WG. The χ test of goodness of t. . *Ann Math Stat* 1952;23:315–345.
36. Higgins JP, Thompson SG. Quantifying heterogeneity in a meta- analysis. . *Statistics in medicine.* 2002;21:1539–1558.
37. Huedo-Medina TB, Sánchez-Meca J, Marín-Martínez F, J. B. Assessing heterogeneity in meta-analysis: Q statistic or I2 index? *Psychol Methods* 2006;11:193–206.
38. Higgins JP, Thompson SG, Spiegelhalter DJ. A re-evaluation of random-effects meta-analysis. . *J R Stat Soc Ser A Stat Soc.* 2009;172:137–159.
39. Sutton AJ, Duval SJ, Tweedie RL, Abrams KR, Jones DR. Empirical assessment of effect of publication bias on meta-analyses. *BMJ.* 2000;320(1574–7).
40. Duval S, Tweedie R. A nonparametric "trim and ll" method of accounting for publication bias in meta-analysis. . *J Am Stat Assoc.* 2000;95:89–98.
41. Egger. M., Davey Smith G, Schneider M, . , Minder C. Bias in meta-analysis detected by a simple, graphical test. . *BMJ* 1997;315:629–634.
42. Loth K, Wall M, Larson N, Neumark-Sztainer D. Disordered eating and psychological well-being in overweight and nonoverweight adolescents: Secular trends from 1999 to 2010. *The International journal of eating disorders.* 2015.
43. Suisman JL, Thompson JK, Keel PK, et al. Genetic and environmental influences on thin-ideal internalization across puberty and preadolescent, adolescent, and young adult development. *The International journal of eating disorders.* 2014;47(7):773-783.
44. Stamatakis E, Wardle J, Cole TJ. Childhood obesity and overweight prevalence trends in England: evidence for growing socioeconomic disparities. *Int J Obes (Lond).* 2010;34(1):41-47.
45. Jaacks LM, Slining MM, Popkin BM. Recent trends in the prevalence of under- and overweight among adolescent girls in low- and middle-income countries. *Pediatric obesity.* 2015.
46. Spencer RA, Rehman L, Kirk SF. Understanding gender norms, nutrition, and physical activity in adolescent girls: a scoping review. *The international journal of behavioral nutrition and physical activity.* 2015;12:6.
47. Goldschmidt AB, Wall MM, Loth KA, Neumark-Sztainer D. Risk Factors for Disordered Eating in Overweight Adolescents and Young Adults. *Journal of pediatric psychology.* 2015;40(10):1048-1055.
48. Atkins D, Best D, Briss PA, et al. Grading quality of evidence and strength of recommendations. . *BMJ* 2004;328:1490–1494.
49. Bogart LM, Elliott MN, Cowgill BO, et al. Two-Year BMI Outcomes From a School-Based Intervention for Nutrition and Exercise: A Randomized Trial. *Pediatrics.* 2016;137(5).
50. Sharif Ishak SI, Chin YS, Mohd Taib MN, Mohd Shariff Z. School-based intervention to prevent overweight and disordered eating in secondary school Malaysian adolescents: a study protocol. *BMC public health.* 2016;16(1):1101.
51. Pbert L, Druker S, Barton B, et al. A School-Based Program for Overweight and Obese Adolescents: A Randomized Controlled Trial. *J Sch Health.* 2016;86(10):699-708.

For peer review only

52. Dunstan CJ, Paxton SJ, McLean SA. An evaluation of a body image intervention in adolescent girls delivered in single-sex versus co-educational classroom settings. *Eating behaviors.* 2016.
53. Datar A, Chung PJ. Accuracy of Weight Perceptions in a Nationally Representative Cohort of US 8th Grade Adolescents. *Academic pediatrics.* 2015.

Authors' contributors

ACBL, the guarantor of the protocol, drafted the protocol and registered it in PROSPERO. TB reviewed and commented on the protocol in PROSPERO. ACBL, DT, KLLD, TN, STP, TL, LAVI and TB all reviewed and commented on this protocol.

Funding statement

This work was supported by the USDA/ARS Children's Nutrition Research Center, Department of Pediatrics, Baylor College of Medicine, Houston, TX under Cooperative Agreement Number 58-6250-6001.

Competing interests statement

No competing of interest

For peer review only

Figure 1 – Scheme of the weight related behaviors

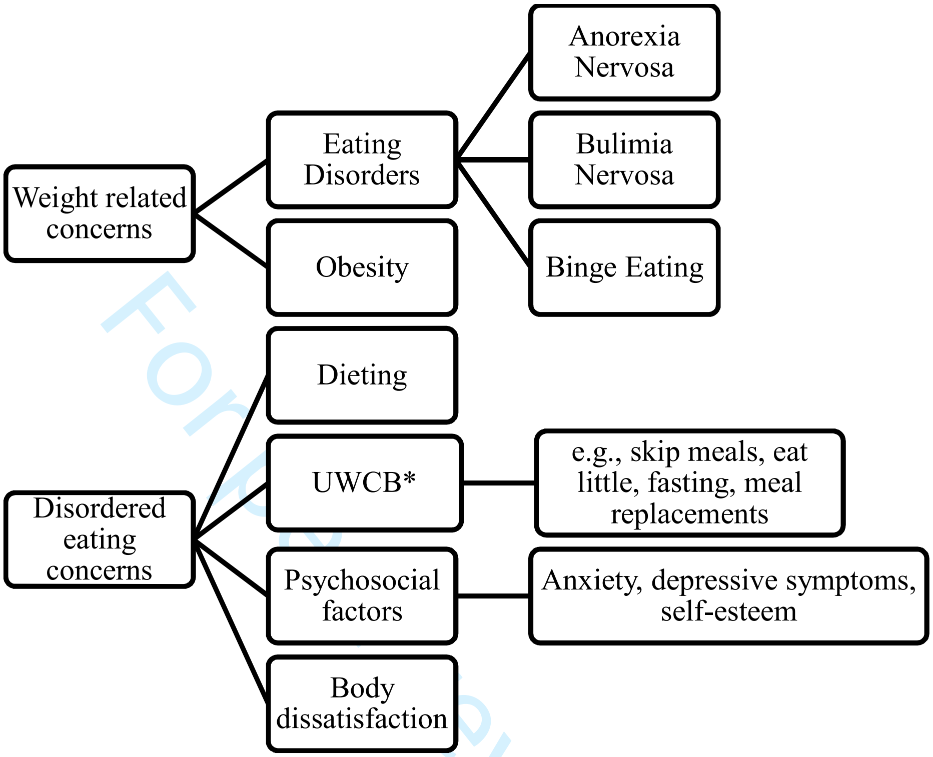

*UWCB = Unhealthy Weight Control Behaviors

For peer review only

PRISMA-P (Preferred Reporting Items for Systematic review and Meta-Analysis Protocols) 2015 checklist: recommended items to address in a systematic review protocol*

Section and topic	Item No	Checklist item	Page
**ADMINISTRATIVE INFORMATION**			
Title:			
Identification	1a	Identify the report as a protocol of a systematic review	1
Update	1b	If the protocol is for an update of a previous systematic review, identify as such	N/A
Registration	2	If registered, provide the name of the registry (such as PROSPERO) and registration number	PROSPERO **CRD42017076547**
Authors:			
Contact	3a	Provide name, institutional affiliation, e-mail address of all protocol authors; provide physical mailing address of corresponding author	1
Contributions	3b	Describe contributions of protocol authors and identify the guarantor of the review	9
Amendments	4	If the protocol represents an amendment of a previously completed or published protocol, identify as such and list changes; otherwise, state plan for documenting important protocol amendments	N/A
Support:			
Sources	5a	Indicate sources of financial or other support for the review	9/10
Sponsor	5b	Provide name for the review funder and/or sponsor	9/10
Role of sponsor or funder	5c	Describe roles of funder(s), sponsor(s), and/or institution(s), if any, in developing the protocol	9/10
**INTRODUCTION**			
Rationale	6	Describe the rationale for the review in the context of what is already known	2 to 5
Objectives	7	Provide an explicit statement of the question(s) the review will address with reference to participants, interventions, comparators, and outcomes (PICO)	5
**METHODS**			
Eligibility criteria	8	Specify the study characteristics (such as PICO, study design, setting, time frame) and report characteristics (such as years considered, language, publication status) to be used as criteria for eligibility for the review	5-6
Information sources	9	Describe all intended information sources (such as electronic databases, contact with study authors, trial registers or other grey literature sources) with planned dates of coverage	6
Search strategy	10	Present draft of search strategy to be used for at least one electronic database, including planned limits,	6

For peer review only

		such that it could be repeated	
Study records:			
Data management	11a	Describe the mechanism(s) that will be used to manage records and data throughout the review	6
Selection process	11b	State the process that will be used for selecting studies (such as two independent reviewers) through each phase of the review (that is, screening, eligibility and inclusion in meta-analysis)	6
Data collection process	11c	Describe planned method of extracting data from reports (such as piloting forms, done independently, in duplicate), any processes for obtaining and confirming data from investigators	7
Data items	12	List and define all variables for which data will be sought (such as PICO items, funding sources), any pre-planned data assumptions and simplifications	6
Outcomes and prioritization	13	List and define all outcomes for which data will be sought, including prioritization of main and additional outcomes, with rationale	6
Risk of bias in individual studies	14	Describe anticipated methods for assessing risk of bias of individual studies, including whether this will be done at the outcome or study level, or both; state how this information will be used in data synthesis	7
Data synthesis	15a	Describe criteria under which study data will be quantitatively synthesised	7-8
	15b	If data are appropriate for quantitative synthesis, describe planned summary measures, methods of handling data and methods of combining data from studies, including any planned exploration of consistency (such as I^2, Kendall's τ)	7-8
	15c	Describe any proposed additional analyses (such as sensitivity or subgroup analyses, meta-regression)	7-8
	15d	If quantitative synthesis is not appropriate, describe the type of summary planned	N/A
Meta-bias(es)	16	Specify any planned assessment of meta-bias(es) (such as publication bias across studies, selective reporting within studies)	N/A
Confidence in cumulative evidence	17	Describe how the strength of the body of evidence will be assessed (such as GRADE)	8

*** It is strongly recommended that this checklist be read in conjunction with the PRISMA-P Explanation and Elaboration (cite when available) for important clarification on the items. Amendments to a review protocol should be tracked and dated. The copyright for PRISMA-P (including checklist) is held by the PRISMA-P Group and is distributed under a Creative Commons Attribution Licence 4.0.**

From: Shamseer L, Moher D, Clarke M, Ghersi D, Liberati A, Petticrew M, Shekelle P, Stewart L, PRISMA-P Group. Preferred reporting items for systematic review and meta-analysis protocols (PRISMA-P) 2015: elaboration and explanation. BMJ. 2015 Jan 2;349(jan02 1):g7647.

For peer review only

BMJ Open

Obesity and eating disorders in integrative prevention programs for adolescents: protocol for a systematic review and meta-analysis

Journal:	*BMJ Open*
Manuscript ID	bmjopen-2017-020381.R1
Article Type:	Protocol
Date Submitted by the Author:	19-Feb-2018

Complete List of Authors:	Barco Leme, Ana Carolina; Baylor College of Medicine, Children's Nutrition Research Center<br>Thompson, Debbe ; Baylor College of Medicine, Children's Nutrition Research Center<br>Lenz Dunker, Karin ; Universidade Federal de Sao Paulo Escola Paulista de Medicina, Department of Psychiatric<br>Nicklas, Theresa; Baylor College of Medicine, Children's Nutrition Research Center<br>Tucunduva Philippi, Sonia ; Universidade de Sao Paulo<br>Lopez, Tabbetha ; University of Houston College of Liberal Arts and Social Sciences, Health and Human Performance<br>Vézina-Im, Lydi-Anne; Baylor College of Medicine, Pediatrics-Nutrition<br>Baranowski, Tom; Baylor College of Medicine, Department of Pediatrics

**Primary Subject Heading**:	Public health
Secondary Subject Heading:	Nutrition and metabolism, Research methods
Keywords:	Adolescent, obesity prevention, Eating disorders < PSYCHIATRY, systematic review, disordered eating

SCHOLARONE™
Manuscripts

Obesity and eating disorders in integrative prevention programs for adolescents: protocol for a systematic review and meta-analysis

Ana Carolina Barco Leme[1]; Debbe Thompson[1], Karin Louise Lenz Dunker[2], Theresa Nicklas[1], Sonia Tucunduva Philippi[3], Tabbetha Lopez[4], Lydi-Anne Vézina-Im[1]; Tom Baranowski[1]

[1] Children's Nutrition Research Center, Baylor College of Medicine, Houston, Texas, USA
[2] Department of Psychiatry, Federal University of São Paulo, São Paulo, Brazil
[3] School of Public Health, University of São Paulo, São Paulo, Brazil
[4] University of Houston, College of Liberal Arts and Social Sciences, Health and Human Performance, Houston, Texas, USA

Corresponding author: Ana Carolina Barco Leme
Children's Nutrition Reseach Center, Baylor College of Medicine.
1100 Bates Street, Houston, TX, 77030-3411, USA.
E-mail: acarol.leme@gmail.com
Telephone: +1(832)209-0406.

Word count: 2710

Abstract for protocol

Introduction: Obesity and eating disorders are public health problems that have lifelong financial and personal costs and common risk factors e.g., body dissatisfaction, weight teasing and disordered eating. Obesity prevention interventions might lead to the development of an eating disorder since focusing on weight may contribute to excessive concern with diet and weight. Therefore, the proposed research will assess whether integrating obesity and eating disorder prevention procedures ("integrated approach") do better than single approach interventions in preventing obesity among adolescents, and if integrated approaches influence weight-related outcomes. **Methods and analysis**: Integrated obesity and eating disorder prevention interventions will be identified. Randomized controlled trials and quasi-experimental trials reporting data on adolescents ranging from 10 to 19 years of age from both sexes will be

For peer review only

included. Outcomes of interest include body composition, unhealthy weight control behaviors, and body satisfaction measurements. MEDLINE/PubMed, PsycINFO, Web of Science and SciELO will be searched. Data will be extracted independently by two reviewers using a standardized data extraction form. Trial quality will be assessed using the Cochrane Collaboration criteria. The effects of integrated vs. single approach intervention studies will be compared using systematic review procedures. If an adequate number of studies report data on integrated interventions among similar populations (k>5), a meta-analysis with random-effects will be conducted. Sensitivity analyses and meta-regression will be performed only if between-study heterogeneity is high ($I^2 \geq 75\%$). **Ethics and dissemination**: Ethics approval will not be required as this is a systematic review of published studies. The findings will be disseminated through conference presentations and peer-reviewed journals.

For peer review only

Strengths and Limitations of this study

- First review and meta-analysis of stand-alone obesity prevention programs vs. integrated obesity and eating disorder prevention approaches on body composition.
- Body composition measures do not precisely measure body fat
- Disordered eating will be measured using self-reported measures
- Age will be limited to 10- to 19-year-old adolescents

Keywords: Adolescent, obesity prevention, eating disorders, systematic review, disordered eating

Background

Pediatric overweight and obesity are worldwide public health concerns[1], with the highest rates in the USA where 28.8% of boys and 29.7% of girls are overweight or obese[2]. Western low- and middle-income countries (LMIC) also face unhealthy child weight, e.g., 24.3% of individuals between 10-19 years of age in Brazil were overweight or obese[2]. Some evidence indicates a rapid increase in prevalence levels in LMICs as high or even higher than those found in high-income countries (HICs)[3]. Obesity has been associated with long and short-term physical

health conditions, such as cardio-metabolic diseases[4], certain types of cancers[5], and mental health concerns[6,7]. Overweight youth are also at high risk of becoming obese adults[8], indicating prevention should be initiated in youth.

Prior systematic reviews have examined childhood obesity prevention studies [3,9,10]. Findings, however, have been mixed. In one review of school-based interventions to prevent obesity among children and adolescents, an average difference between the intervention and control groups was -0.33kg/m^2 (-0.55, -0.11, 95%CI), with 84% of this effect explained by the highest quality studies[11]. Alternatively, another reported a difference of 0.03 (95%CI: 0.09 to 0.03, p=0.03) with high heterogeneity (I^2=87%) [12]. Thus, evidence regarding the effectiveness of school-based obesity prevention interventions to reduce BMI in youth is mixed with high heterogeneity among studies. Narrower age groups who experience common problems and receive interventions appropriate to these common problems may be more effective.

Eating disorders are illnesses in which the people experience severe disturbances in their eating behaviors and related thoughts and emotions. People with eating disorders typically become pre-occupied with food and their body weight[13]. In the Diagnostic and Statistical Manual of Mental Disorders 5, the eating disorders section was renamed “Feeding and Eating Disorders” and specified three eating disorders: anorexia nervosa, bulimia nervosa and binge eating disorder; and three feeding disorders: pica, rumination disorder, and avoidant/restrictive food disorder[13]. These categories, and associated criteria served to decrease the frequency of the diagnostic category “eating disorder not otherwise specified”, a heterogeneous not well-defined group of eating disorders. Eating disorder not otherwise specified was the most common diagnosis in clinical and community samples of adolescents, accounting for around 80% of all eating disorder diagnoses, with psychopathology and adverse consequences comparable to anorexia nervosa and bulimia nervosa[13,14].

Disordered eating behaviors and attitudes are part of the eating disorders continuum and include obsessively thinking about food and calories, becoming angry when hungry, being unable to select what to eat, seeking food to compensate for psychological problems, eating until feeling sick, and presenting unreal myths and beliefs about eating and weight[15]. Disordered eating is not limited to those diagnosed with eating disorders. Indeed, many individuals experience disordered eating behaviors, beliefs, and feelings toward food but are unaware that they are manifesting “abnormal” behaviors[16].

For peer review only

Most interventions in the field of eating disorders can be classified as primary prevention programs, aiming to reduce risk factors. In general, these interventions focus on girls as a target group based on the observation, that girls have an increased chance of developing an eating disorder, especially anorexia and bulimia nervosa[17]. Schools are the most common setting for the existing evaluated programs[17]. Earlier eating disorder programs tended to employ fear appeals, threat appeals or fear arousing communications[17,18]. These methods have been increasingly abandoned, since they did not show an effect or might have even been "more harmful than beneficial" [17]. More recent eating disorder prevention programs focused on protective factors such as life skills and emotion regulation competence[19,20]. The PriMa (Primary prevention of Anorexia Nervosa) program [17], was a new type of prevention program for girls up to the age of 12. This scientifically based intervention attempted to prevent eating disorders and reduce disordered eating behaviors by primarily focusing on problems associated with anorexia nervosa. The nine lesson-program utilized standardized posters and guidelines to encourage group discussions. The intervention group reported significant improvements in body self-esteem, figure dissatisfaction, knowledge and eating attitudes. Also, instead of interventionists, the program used school teachers to deliver the intervention.

A recent systematic review and meta-analyses [20] quantified the effectiveness of eating disorder preventive randomized controlled trials for children, adolescents and youth. A total of 112 studies were included; 58% of the trials had high risk of bias. Findings indicated small to moderate effect sizes in reducing eating disorder risk factors. It also revealed that promising preventive interventions for eating disorders risk factors may include cognitive dissonance therapy, cognitive behavioral therapy and media literacy. Whether these interventions lower eating disorder incidence is, however, uncertain, there is a need for studies that combine eating disorder and obesity prevention [20].

Although eating disorder prevention programs included content of relevance to obesity prevention (e.g., promotion of healthy weight management) few assessed the impact on weight status or other obesity-related outcomes[18,20,21].

Being overweight during childhood increases the chances of having an eating disorder during adulthood (compared to normal weight controls) [17]. Common obesity and eating disorders risk factors can be categorized into three levels according to the Social Ecological Model [22]: individual (e.g., sex, age and weight-status), social (e.g., media, weight teasing and ideal beauty

For peer review only

pattern) and psychological (e.g., self-esteem and body satisfaction). Several studies have described the co-presence of these factors, which could be considered risks for the development of eating disorders and obesity[23-25]. Thus, an integrated approach could address the differences in these prevention philosophies, e.g., eating behaviors (dieting vs. no dieting) and body weight (lose vs. accept weight)[19].

Some interventions addressed both obesity and eating disorders in prevention interventions because of the efficiency in addressing two conditions with a single intervention and a possible reduced risk of inadvertently causing eating disorders while trying to prevent obesity[26-28], e.g., strategies to prevent obesity (monitoring intake and portion control) might unintentionally promote shape concerns and disordered eating. Integrating obesity and eating disorder prevention programs may prove easier and more cost-effective than treating them separately, and healthy nutrition and physical activity are the focus of both eating disorders and obesity prevention programs[29]. Body dissatisfaction concerns were also addressed in both approaches, but have mismatched messages. For example, some obesity prevention programs considered it acceptable to be unhappy about being overweight in order to motivate restricting the amount and content of food consumed to reduce body weight[29], while eating disorder prevention programs promoted self-acceptance at any weight, discouraging self-consciousness about dietary intake. However, data supporting these alternative viewpoints are scarce [29].

Due to the increasing prevalence of obesity and eating disorders [21,26] and shared common risk factors, i.e., body dissatisfaction, unhealthy weight control behaviors/dieting and weight teasing (Figure 1) there have been calls for integration to address these common concerns[26]. For instance, obesity and eating disorders can co-occur in the same individual[26]. A cross-cultural comparison between US and Spanish adolescents found dieting and use of unhealthy weight control behaviors were higher among overweight and obese youth and concluded that prevention interventions should address the broad spectrum of eating and weight-related problems [30].

In summary, obesity and eating disorders have common risk factors with adverse health outcomes, mainly among overweight and female adolescents. Systematic reviews and meta-analyses have only analyzed results for single approaches (i.e., obesity or eating disorders prevention) and the results have been mixed. Interventions that integrate obesity and eating disorders prevention components might be more effective. A review of such interventions might provide insight into the mechanisms of effect and inform interventions that address both

For peer review only

problems simultaneously. To the authors' knowledge, no previous review has identified the impact of integrated obesity and eating disorders prevention programs for adolescents. The present systematic review will answer the following questions:

- Do integrated obesity and eating disorders interventions do better than obesity-only prevention interventions in improving adolescents' health behavior outcomes and maintaining healthy weight status?
- Do integrated interventions promote being more satisfied with one's body and reduce unhealthy weight control behaviors in adolescents?

Methods and analysis

The study protocol was accepted by PROSPERO (www.crd.york.ac.uk/PROSPERO) in October 2017 (CRD42017076547). This protocol follows the Preferred Reporting Items for Systematic Review and Meta-Analysis Protocol (PRISMA-P) checklist[31]. Modifications to the protocol will be tracked and dated in PROSPERO.

Patient and public involvement

Patients and or public were not involved in this current study

Criteria for considering studies for this review

Inclusion criteria

Population: adolescents 10 to 19 years of age from both sexes. Adolescents in this age range are at increased risk for unhealthy weight control behaviors and body satisfaction, shared risk factors for obesity and eating disorders[19,32]. Most published integrated prevention studies are in this age group.

Type of outcomes: (1) body composition measurements (i.e., body mass index (BMI), waist circumference or percent body fat); (2) weight control behaviors and/or scales that assess the risk for an eating disorders (such as the Eating Attitudes Test (EAT-26), Sociocultural Attitudes Towards Appearance Questionnaire 3 (SATAQ-3) and Eating Disorder Examination Questionnaire (EDE-Q)); (3) self-reported scales on body satisfactions and; (4) other psychological markers (e.g., anxiety, depression and/or self-esteem inventories). Inclusion of at

For peer review only - http://bmjopen.bmj.com/site/about/guidelines.xhtml

least one of the weight control behaviors and/or scales must have been used to assess the risk for eating disorders [16,19,33,34].

We define “obesity and eating disorder prevention studies” to be those in which the authors explicitly state they are targeting both sets of outcomes. “Obesity prevention alone” studies are defined to be those in which the authors state only an obesity prevention objective even if mentioning eating disorder prevention. Some obesity prevention studies collect measures of eating disorders to assess possible unanticipated eating disorder side effects. These will be considered obesity prevention alone studies.

Study design: Quasi-randomized controlled trials and randomized controlled trials assessing the impact of integrated or obesity- only prevention interventions.

Type of studies: Quantitative outcome analyses will be included in the systematic review and meta-analysis.

Search strategy

A structured electronic search will employ all publication years (up to 2018) using four databases and terms will be searched for all text: Medical Literature Library of Medicine (MEDLINE) via PubMed (≥1979), PsycINFO of the American Psychological Association (≥1954), Web of Science via Clarivate Analytics (≥1983) and Scientific Electronic Library (SciELO) via BIREME Latin American and Caribbean Center on Health Science Information (≥1997). Systematic searchers will be developed from this model, applied in MEDLINE: (Obesity) OR Overweight) OR Weight related problems) AND eating disorder) OR weight control behaviors) AND adolescents) OR youth) OR teenagers) OR girls) OR boys) AND prevent*) OR strategies) OR randomized controlled trial. Congress abstracts, dissertations, theses, and articles published in journals without peer-review will not be included in the review. Only studies written in English, German, Spanish or Portuguese will be included. The results of this search strategy will be reported in a PRISMA flowchart[31]. The bibliographies of papers that match inclusion criteria will be searched by hand to identify further relevant references, which will be subjected to the same screening and selection process. The full search strategy is referred in the supplementary file (Supplement figure 1).

For peer review only

Screening and data extraction

All articles identified from the initial electronic search process will be imported into an Endnote library, and duplicates removed. The eligibility criteria will be applied to the results and all identified references screened independently by two reviewers (AL and TL) in a standard blinded way in four stages: (i) reviewing the titles and abstracts; (ii) retrieving and examining the full texts for inclusion; (iii) searching references lists from the full articles; and (iv) examining relevant references for additional studies. TB will be consulted when questions or ambiguity arise. The data extraction form will be pre-tested with 5 randomly selected trials.

Quality assessment

The quality of the randomized controlled trials will be assessed using the Cochrane Collaboration's tool for assessing risk of bias in randomized trials [35]. All data will be extracted and quality assessed by two reviewers. Disagreements at each step will be resolved by discussion. When no consensus is reached a third reviewer will resolve the discrepancy.

Data synthesis and analysis

The results of the studies included in the systematic review will be described in a summary table, consisting of author (year), purpose of the study, population targeted, study quality[35], characteristics of the sample, outcome measures, statistical analyses performed (e.g., repeated measure ANOVA, ANCOVA or regression analysis) and the results on body composition and disordered eating behaviors. The results of the impact of the intervention will be reported in effect sizes, such as Odd Ratios (OR) for dichotomous outcomes (e.g., satisfied and dissatisfied) or standardized mean differences (SMD) for continuous outcomes (e.g., BMI – Kg/m^2). All effect sizes will be zero order. To facilitate interpretation and permit comparison with other SMD and standard effect sizes, the ORs will be converted to Cohen's d[36]. Cohen's d of 0.2 is a small effect size, 0.5 is medium and ≥0.80 is large[36].

An adequate number of studies ($k>5$)[37] will trigger a meta-analysis of the findings with a random-effects model. The magnitude of the effect sizes might vary across the studies due to the differences in sample and outcomes of the studies. The pooled effect sizes will be computed, and each study will be weighted according to its sample size. Cochran's Q[38] and I^2 statistics[39] will assess the between-study heterogeneity, as measures of the percentage of total variation in estimated effects that is a consequence of heterogeneity rather than chance[40]. Significant

For peer review only

heterogeneity is considered when the Q statistic has $p<0.05$. An I^2 statistic of 25% or less is considered low; 50% moderate and 75% high heterogeneity[41]. If study heterogeneity exceeds $I^2 \geq 75\%$ (high), it will be explored through sensitivity analyses and meta-regression. The funnel plot will be inspected for publication bias, with a minimum of 10 studies in the analysis[42], through Duval and Tweedie's trim and fill method[43] and Egger's regression test[44]. All the analyses will be conducted using Comprehensive Meta-Analysis software.

Subgroup analyses might be conducted to assess the possible effects of time differences between integrated prevention versus single obesity approach, and between certain disordered eating behaviors and anthropometric measurements according to the following variables: population (e.g., normal weight adolescents vs. overweight/obese adolescents) and quality rating (high-rated vs. low-rated studies according to the Cochrane Collaboration's tool[35]). Age and sex differences in the impact of integrated prevention programs will be examined since disorder eating behaviors are more common among older adolescents, girls and overweight/obese individuals[26,45,46]. Moreover, because previous studies[47-50] have found socioeconomic disparities in obesity and disorder eating socioeconomic status, differences will be examined in eating disorder and obesity risk factors in the prevention conditions.

The strength of the evidence will be evaluated using the Grading of Recommendations Assessment, Development and Evaluation (GRADE) guidelines[51]. The following assessments will be made: (1) Quality rating for each study according to Cochrane Collaboration's tool[35]; (2) Cohen's d classification to evaluate the magnitude of individual or pooled effect size (SMD)[36], if a meta-analysis is possible; (3) Cochran's Q[38] and I^2 statistic[39] for heterogeneity; and (4) risk of bias by visualizing the distribution of the funnel plot if there are at least 10 trials per analysis[42] through Duval and Tweedie's trim and fill method and Eggers's regression test.

Gaps and limitations

Several gaps and limitations should be noted in anticipation of the findings of the systematic review and meta-analysis. First, the body composition measurements reported in these studies will always be objective measurements (i.e., BMI, waist circumference and %body composition) which do not precisely measure percentage body fat[52-54]. Second, eating disorder will be assessed through self-reported measurements which might provide biased responses, since underreporting is highly prevalent, especially among girls and overweight/obese

For peer review only

individuals[45,49,50,55,56]. Any studies that assess disease preventive intervention impacts on self-reported or anthropometric data may potentially underestimate the effect of the intervention[19]. Finally, we are going to cover only adolescents aging from 10 to 19 years old. However, the majority of the integrated interventions focus on these adolescent years[19].

Implications

This systematic review and meta-analysis aims to evaluate the impact of obesity and eating disorder prevention programs for adolescents. Results of this study should provide new insights into the approaches tested thus far. The systematic review and meta-analysis may also identify specific gaps in the evidence, which would inform the agenda for future research and policy.

Amendments

If there is a need to amend this protocol, the date, rationale, and a description of each protocol change will be reported.

Ethics and dissemination

Ethics approval will not be required as this is a protocol for systematic review and meta-analysis. This systematic review and meta-analysis will be published in a peer-reviewed journal which will be disseminated electronically and in print.

Abbreviations

EPHPP: Effective Public Health Project; GRADE: Grading of Recommendations Assessment Development and Evaluation; HIC: High-income countries; LMIC: Low- middle-income countries.

Contributors

ACBL, the guarantor of the protocol, drafted the protocol and registered it in PROSPERO. TB reviewed and commented on the protocol in PROSPERO. ACBL, DT, KLLD, TN, STP, TL, LAVI and TB all reviewed and commented on this protocol.

For peer review only

Funding

This work is a publication of the USDA (USDA/ARS) Children's Nutrition Research Center, Department of Pediatrics, Baylor College of Medicine (Houston, TX) and has had been funded, in part, with federal funds from the USDA/ARS under Cooperative Agreement Number 58-6250-6001. ACBL received a postdoctoral fellowship from the State of São Paulo, Brazil (FAPESP process nº 2016/ 21144-9).

Competing interests

None declared.

Figure legend

*UWCB = Unhealthy Weight Control Behaviors

References

1. Hoelscher DM, Kirk S, Ritchie L, Cunningham-Sabo L, Academy Positions C. Position of the Academy of Nutrition and Dietetics: interventions for the prevention and treatment of pediatric overweight and obesity. *Journal of the Academy of Nutrition and Dietetics.* 2013;113(10):1375-1394.
2. Ng M, Fleming T, Robinson M, et al. Global, regional, and national prevalence of overweight and obesity in children and adults during 1980–2013: a systematic analysis for the Global Burden of Disease Study 2013. *The Lancet.* 2014;384(9945):766-781.
3. Verstraeten R, Roberfroid D, Lachat C, et al. Effectiveness of preventive school-based obesity interventions in low- and middle-income countries: a systematic review. *The American journal of clinical nutrition.* 2012;96(2):415-438.
4. Moreno LA. Obesity in children and adolescents. A critical review. *Endocrinología y Nutrición.* 2013;60:7-9.
5. Kann L, McManus T, Harris WA, et al. Youth Risk Behavior Surveillance - United States, 2015. *MMWR Surveill Summ.* 2016;65(6):1-174.
6. Reilly JJ, Kelly J. Long-term impact of overweight and obesity in childhood and adolescence on morbidity and premature mortality in adulthood: systematic review. *Int J Obes (Lond).* 2011;35(7):891-898.
7. Wake M, Canterford L, Patton GC, et al. Comorbidities of overweight/obesity experienced in adolescence: longitudinal study. *Archives of disease in childhood.* 2010;95(3):162-168.

For peer review only

8. Tsiros MD, Coates AM, Howe PR, Grimshaw PN, Buckley JD. Obesity: the new childhood disability? *Obesity reviews : an official journal of the International Association for the Study of Obesity.* 2011;12(1):26-36.
9. Wang Y, Cai L, Wu Y, et al. What childhood obesity prevention programmes work? A systematic review and meta-analysis. *Obes Rev.* 2015;16(7):547-565.
10. Boff RM, Liboni RPA, Batista IPA, de Souza LH, Oliveira MDS. Weight loss interventions for overweight and obese adolescents: a systematic review. *Eating and weight disorders : EWD.* 2017;22(2):211-229.
11. Silveira JA, Taddei JA, Guerra PH, Nobre MR. The effect of participation in school-based nutrition education interventions on body mass index: a meta-analysis of randomized controlled community trials. *Preventive medicine.* 2013;56(3-4):237-243.
12. Guerra PH, Nobre MR, da Silveira JA, Taddei JA. School-based physical activity and nutritional education interventions on body mass index: a meta-analysis of randomised community trials - project PANE. *Preventive medicine.* 2014;61:81-89.
13. APA. *Diagnostic and Statistical Manual of Mental Disorders (DSM-5®).* Arlington, VA: American Psychiatric Association;2013.
14. Smink FR, van Hoeken D, Oldehinkel AJ, Hoek HW. Prevalence and severity of DSM-5 eating disorders in a community cohort of adolescents. *Int J Eat Disord.* 2014;47(6):610-619.
15. Alvarenga Mde F, Scagliusi F, Philippi ST. Development and validity of the Disordered Eating Attitude Scale (DEAS). *Perceptual and motor skills.* 2010;2010(110):379-395.
16. Alvarenga Mdos S, Scagliusi FB, Philippi ST. Development and validity of the Disordered Eating Attitude Scale (DEAS). *Percept Mot Skills.* 2010;110(2):379-395.
17. Berger U, Sowa M, Bormann B, Brix C, Strauss B. Primary prevention of eating disorders: characteristics of effective programmes and how to bring them to broader dissemination. *European eating disorders review : the journal of the Eating Disorders Association.* 2008;16(3):173-183.
18. Stice E, Shaw H, Marti CN. A meta-analytic review of eating disorder prevention programs: encouraging findings. *Annu Rev Clin Psychol.* 2007;3:207-231.
19. Sanchez-Carracedo D, Neumark-Sztainer D, Lopez-Guimera G. Integrated prevention of obesity and eating disorders: barriers, developments and opportunities. *Public health nutrition.* 2012;15(12):2295-2309.
20. Le LK, Barendregt JJ, Hay P, Mihalopoulos C. Prevention of eating disorders: A systematic review and meta-analysis. *Clinical psychology review.* 2017;53:46-58.
21. Leal GVS, Philippi ST, Polacow VO, Cordas TA, Alvarenga Mdos S. O que é comportamento de risco para transtornos alimentares em adolescentes? *Jornal Brasileiro de Psiquiatria.* 2013;62 (1):62-75.
22. Sallis JF, Owen N, Fisher EB. Ecological models of health behavior, in Health behavior and health education. Theory, research, and practice. . In: San Francisco, CA: Jossey-Bass; 2008:465–485.
23. Balantekin KN, Birch LL, Savage JS. Family, friend, and media factors are associated with patterns of weight-control behavior among adolescent girls. *Eat Weight Disord.* 2017.

For peer review only

24. Frederick DA, Sandhu G, Scott T, Akbari Y. Reducing the negative effects of media exposure on body image: Testing the effectiveness of subvertising and disclaimer labels. *Body image.* 2016;17:171-174.
25. Loth K, Wall M, Larson N, Neumark-Sztainer D. Disordered eating and psychological well-being in overweight and nonoverweight adolescents: secular trends from 1999 to 2010. *The International journal of eating disorders.* 2015;48(3):323-327.
26. Haines J, Neumark-Sztainer D. Prevention of obesity and eating disorders: a consideration of shared risk factors. *Health Educ Res.* 2006;21(6):770-782.
27. Tanofsky-Kraff M, Shomaker LB, Wilfley DE, et al. Targeted prevention of excess weight gain and eating disorders in high-risk adolescent girls: a randomized controlled trial. *Am J Clin Nutr.* 2014;100(4):1010-1018.
28. Sanchez-Carracedo D, Fauquet J, Lopez-Guimera G, et al. The MABIC project: An effectiveness trial for reducing risk factors for eating disorders. *Behav Res Ther.* 2016;77:23-33.
29. Irving LM, Neumark-Sztainer D. Integrating the prevention of eating disorders and obesity: feasible or futile? *Preventive medicine.* 2002;34(3):299-309.
30. Lopez-Guimera G, Neumark-Sztainer D, Hannan P, Fauquet J, Loth K, Sanchez-Carracedo D. Unhealthy weight-control behaviours, dieting and weight status: a cross-cultural comparison between North American and Spanish adolescents. *Eur Eat Disord Rev.* 2013;21(4):276-283.
31. Moher D, Liberati A, Tetzlaff J, Altman DG, Group P. Preferred reporting items for systematic reviews and meta-analyses: the PRISMA statement. *PLoS medicine.* 2009;6(7):e1000097.
32. Watson HJ, Joyce T, French E, et al. Prevention of eating disorders: A systematic review of randomized, controlled trials. *The International journal of eating disorders.* 2016;49(9):833-862.
33. Neumark-Sztainer D. Integrating messages from the eating disorders field into obesity prevention. *Adolesc Med State Art Rev.* 2012;23(3):529-543.
34. Neumark-Sztainer D. Addressing obesity and other weight-related problems in youth. *Arch Pediatr Adolesc Med.* 2005;159(3):290-291.
35. Higgins JP, Altman DG, Gotzsche PC, et al. The Cochrane Collaboration's tool for assessing risk of bias in randomised trials. *BMJ.* 2011;343:d5928.
36. Cohen J. A power primer. *Psychol Bull* 1992;112:155–159.
37. Davey J, Turner RM, Clarke MJ, Higgins PT. Characteristics of meta-analyses and their component studies in the Cochrane Database of Systematic Reviews: a cross-sectional, descriptive analysis. *BMC public health.* 2011;11.
38. Cochran WG. The χ test of goodness of t. . *Ann Math Stat* 1952;23:315–345.
39. Higgins JP, Thompson SG. Quantifying heterogeneity in a meta- analysis. . *Statistics in medicine.* 2002;21:1539–1558.
40. Huedo-Medina TB, Sánchez-Meca J, Marín-Martínez F, J. B. Assessing heterogeneity in meta-analysis: Q statistic or I2 index? *Psychol Methods* 2006;11:193–206.
41. Higgins JP, Thompson SG, Spiegelhalter DJ. A re-evaluation of random-effects meta-analysis. . *J R Stat Soc Ser A Stat Soc.* 2009;172:137–159.
42. Sutton AJ, Duval SJ, Tweedie RL, Abrams KR, Jones DR. Empirical assessment of effect of publication bias on meta-analyses. *BMJ.* 2000;320(1574–7).

For peer review only

43. Duval S, Tweedie R. A nonparametric “trim and ll” method of accounting for publication bias in meta-analysis. . *J Am Stat Assoc.* 2000;95:89–98.
44. Egger. M., Davey Smith G, Schneider M, . , Minder C. Bias in meta-analysis detected by a simple, graphical test. . *BMJ* 1997;315:629–634.
45. Loth K, Wall M, Larson N, Neumark-Sztainer D. Disordered eating and psychological well-being in overweight and nonoverweight adolescents: Secular trends from 1999 to 2010. *The International journal of eating disorders.* 2015.
46. Suisman JL, Thompson JK, Keel PK, et al. Genetic and environmental influences on thin-ideal internalization across puberty and preadolescent, adolescent, and young adult development. *The International journal of eating disorders.* 2014;47(7):773-783.
47. Stamatakis E, Wardle J, Cole TJ. Childhood obesity and overweight prevalence trends in England: evidence for growing socioeconomic disparities. *Int J Obes (Lond).* 2010;34(1):41-47.
48. Jaacks LM, Slining MM, Popkin BM. Recent trends in the prevalence of under- and overweight among adolescent girls in low- and middle-income countries. *Pediatric obesity.* 2015.
49. Spencer RA, Rehman L, Kirk SF. Understanding gender norms, nutrition, and physical activity in adolescent girls: a scoping review. *The international journal of behavioral nutrition and physical activity.* 2015;12:6.
50. Goldschmidt AB, Wall MM, Loth KA, Neumark-Sztainer D. Risk Factors for Disordered Eating in Overweight Adolescents and Young Adults. *Journal of pediatric psychology.* 2015;40(10):1048-1055.
51. Atkins D, Best D, Briss PA, et al. Grading quality of evidence and strength of recommendations. . *BMJ* 2004;328:1490–1494.
52. Bogart LM, Elliott MN, Cowgill BO, et al. Two-Year BMI Outcomes From a School-Based Intervention for Nutrition and Exercise: A Randomized Trial. *Pediatrics.* 2016;137(5).
53. Sharif Ishak SI, Chin YS, Mohd Taib MN, Mohd Shariff Z. School-based intervention to prevent overweight and disordered eating in secondary school Malaysian adolescents: a study protocol. *BMC public health.* 2016;16(1):1101.
54. Pbert L, Druker S, Barton B, et al. A School-Based Program for Overweight and Obese Adolescents: A Randomized Controlled Trial. *J Sch Health.* 2016;86(10):699-708.
55. Dunstan CJ, Paxton SJ, McLean SA. An evaluation of a body image intervention in adolescent girls delivered in single-sex versus co-educational classroom settings. *Eating behaviors.* 2016.
56. Datar A, Chung PJ. Accuracy of Weight Perceptions in a Nationally Representative Cohort of US 8th Grade Adolescents. *Academic pediatrics.* 2015.

For peer review only

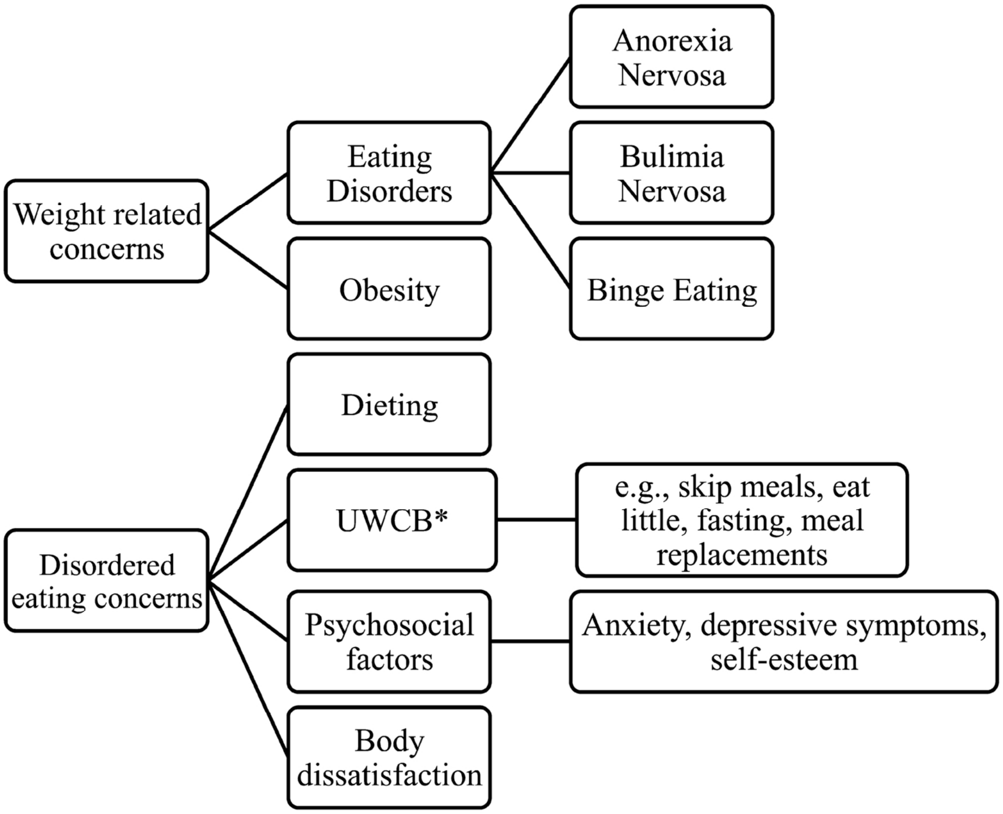

Figure 1 – Scheme of the weight-related behaviors

73x59mm (600 x 600 DPI)

Supplement figure 1 – Search strategy in MedLine

exp "Feeding and eating disorders"/	27,825
(bing* and (food or eat*)).ti.	1,963
(bing* and (food or eat*)).ab.	5,375
(bing* and (food or eat*)).kw.	199
(disorder* and (food or eat*)).ti.	9,465
(disorder* and (food or eat*)).ab.	28,521
(disorder* and (food or eat*)).kw.	72
(anorexia or anorexic).ti,ab,kw.	28,138
bulimi* or bulemi*).ti,ab,kw.	7,825
1 or 2 or 3 or 4 or 5 or 6 or 7 or 8 or 9	63,512
exp Obesity/	183,262
exp Overweight/	188,264
exp Body Weight/	422,550
obes*.ti,ab,kw.	247,930
(overweight or "over weight").ti,ab,kw.	55,791
11 or 12 or 13 or 14 or 15	528,049
exp Primary Prevention/	138,711
"prevention & control".fs.	1,201,380
exp Health Promotion/	68,683
exp Health Education/	156,404
exp School Health Services/	22,370
prevent*.ti,ab,kw.	1,207,081
educat*.ti,ab,kw.	497,253
promot*.ti,ab,kw.	812,498
17 or 18 or 19 or 20 or 21 or 22 or 23 or 24	3,303,119
exp Adolescent/	1,884,302
(adolescen* or teen* or youth*).ti,ab,kw.	296,306
26 or 27	1,955,018
10 and 6 and 25 and 28	982

PRISMA-P (Preferred Reporting Items for Systematic review and Meta-Analysis Protocols) 2015 checklist: recommended items to address in a systematic review protocol*

Section and topic	Item No	Checklist item	Page
**ADMINISTRATIVE INFORMATION**			
Title:			
Identification	1a	Identify the report as a protocol of a systematic review	1
Update	1b	If the protocol is for an update of a previous systematic review, identify as such	N/A
Registration	2	If registered, provide the name of the registry (such as PROSPERO) and registration number	PROSPERO **CRD42017076547**
Authors:			
Contact	3a	Provide name, institutional affiliation, e-mail address of all protocol authors; provide physical mailing address of corresponding author	1
Contributions	3b	Describe contributions of protocol authors and identify the guarantor of the review	10
Amendments	4	If the protocol represents an amendment of a previously completed or published protocol, identify as such and list changes; otherwise, state plan for documenting important protocol amendments	N/A
Support:			
Sources	5a	Indicate sources of financial or other support for the review	10-11
Sponsor	5b	Provide name for the review funder and/or sponsor	10-11
Role of sponsor or funder	5c	Describe roles of funder(s), sponsor(s), and/or institution(s), if any, in developing the protocol	10-11
**INTRODUCTION**			
Rationale	6	Describe the rationale for the review in the context of what is already known	2-6
Objectives	7	Provide an explicit statement of the question(s) the review will address with reference to participants, interventions, comparators, and outcomes (PICO)	6
**METHODS**			
Eligibility criteria	8	Specify the study characteristics (such as PICO, study design, setting, time frame) and report characteristics (such as years considered, language, publication status) to be used as criteria for eligibility for the review	6-7
Information sources	9	Describe all intended information sources (such as electronic databases, contact with study authors, trial registers or other grey literature sources) with planned dates of coverage	7
Search strategy	10	Present draft of search strategy to be used for at least one electronic database, including planned limits,	7

For peer review only

		such that it could be repeated	
Study records:			
Data management	11a	Describe the mechanism(s) that will be used to manage records and data throughout the review	7-8
Selection process	11b	State the process that will be used for selecting studies (such as two independent reviewers) through each phase of the review (that is, screening, eligibility and inclusion in meta-analysis)	7-8
Data collection process	11c	Describe planned method of extracting data from reports (such as piloting forms, done independently, in duplicate), any processes for obtaining and confirming data from investigators	7-8
Data items	12	List and define all variables for which data will be sought (such as PICO items, funding sources), any pre-planned data assumptions and simplifications	6
Outcomes and prioritization	13	List and define all outcomes for which data will be sought, including prioritization of main and additional outcomes, with rationale	6
Risk of bias in individual studies	14	Describe anticipated methods for assessing risk of bias of individual studies, including whether this will be done at the outcome or study level, or both; state how this information will be used in data synthesis	8
Data synthesis	15a	Describe criteria under which study data will be quantitatively synthesised	8
	15b	If data are appropriate for quantitative synthesis, describe planned summary measures, methods of handling data and methods of combining data from studies, including any planned exploration of consistency (such as I^2, Kendall's τ)	8-9
	15c	Describe any proposed additional analyses (such as sensitivity or subgroup analyses, meta-regression)	8-9
	15d	If quantitative synthesis is not appropriate, describe the type of summary planned	N/A
Meta-bias(es)	16	Specify any planned assessment of meta-bias(es) (such as publication bias across studies, selective reporting within studies)	N/A
Confidence in cumulative evidence	17	Describe how the strength of the body of evidence will be assessed (such as GRADE)	9

*** It is strongly recommended that this checklist be read in conjunction with the PRISMA-P Explanation and Elaboration (cite when available) for important clarification on the items. Amendments to a review protocol should be tracked and dated. The copyright for PRISMA-P (including checklist) is held by the PRISMA-P Group and is distributed under a Creative Commons Attribution Licence 4.0.**

From: Shamseer L, Moher D, Clarke M, Ghersi D, Liberati A, Petticrew M, Shekelle P, Stewart L, PRISMA-P Group. Preferred reporting items for systematic review and meta-analysis protocols (PRISMA-P) 2015: elaboration and explanation. BMJ. 2015 Jan 2;349(jan02 1):g7647.

For peer review only
